# Comparative Transcriptomic Analysis of the Hematopoietic System between Human and Mouse by Single Cell RNA Sequencing

**DOI:** 10.3390/cells10050973

**Published:** 2021-04-21

**Authors:** Shouguo Gao, Zhijie Wu, Jeerthi Kannan, Liza Mathews, Xingmin Feng, Sachiko Kajigaya, Neal S. Young

**Affiliations:** Hematology Branch, NHLBI, National Institutes of Health, Bethesda, MD 20892, USA; zhijie.wu@nih.gov (Z.W.); jeerthi.kannan@nih.gov (J.K.); liza.mathews@nih.gov (L.M.); xingmin.feng@nih.gov (X.F.); sachiko.kajigaya@nih.gov (S.K.); youngns@nhlbi.nih.gov (N.S.Y.)

**Keywords:** hematopoiesis, gene regulatory network, single-cell RNA sequencing, cross-species analysis

## Abstract

(1) Background: mouse models are fundamental to the study of hematopoiesis, but comparisons between mouse and human in single cells have been limited in depth. (2) Methods: we constructed a single-cell resolution transcriptomic atlas of hematopoietic stem and progenitor cells (HSPCs) of human and mouse, from a total of 32,805 single cells. We used Monocle to examine the trajectories of hematopoietic differentiation, and SCENIC to analyze gene networks underlying hematopoiesis. (3) Results: After alignment with Seurat 2, the cells of mouse and human could be separated by same cell type categories. Cells were grouped into 17 subpopulations; cluster-specific genes were species-conserved and shared functional themes. The clustering dendrogram indicated that cell types were highly conserved between human and mouse. A visualization of the Monocle results provided an intuitive representation of HSPC differentiation to three dominant branches (Erythroid/megakaryocytic, Myeloid, and Lymphoid), derived directly from the hematopoietic stem cell and the long-term hematopoietic stem cells in both human and mouse. Gene regulation was similarly conserved, reflected by comparable transcriptional factors and regulatory sequence motifs in subpopulations of cells. (4) Conclusions: our analysis has confirmed evolutionary conservation in the hematopoietic systems of mouse and human, extending to cell types, gene expression and regulatory elements.

## 1. Introduction

Hematopoiesis is a stepwise process originating from hematopoietic stem cells (HSCs) associated, functionally, with loss of self-renewal, activation of lineage specific transcription factors (TFs), and upregulation of downstream genes for progenitor cells and their mature progenies [1,2,3]. From multipotent progenitors, the common lineages for myelopoiesis (common myeloid progenitor, CMP) and lymphopoiesis (common lymphoid progenitor, CLP) are segregated. During myeloid differentiation, oligopotent CMPs undergo further restriction into bivalent granulocyte–monocyte progenitors (GMPs) of granulocytes and monocytes, and megakaryocyte–erythroid progenitors (MEPs) that terminate in platelets and red blood cells [4]. The classical model of hematopoiesis was a tree, rooted in the long-term hematopoietic stem cell (LTHSC) and branching into multipotent, oligopotent, and unipotent progenitors. However, novel single cell approaches, which can profile gene expression of thousands of individual cells, have challenged established models of hematopoiesis [5,6].

Single cell RNA-sequencing (scRNA-seq) has been used to examine the conservation of transcription across species in various organs [7,8,9,10,11]. For example, scRNA-seq revealed the surprisingly well-conserved cellular architecture between human and mouse brains: similarity in hierarchical organization, corresponding relationships at the subclass level and no major missing homologous classes [10]. In pancreas, scRNA-seq analysis showed major cell types (alpha, beta, gamma, delta, ductal and endothelial) to be conserved between mouse and human [7]. Shay T et al. observed that the expression patterns of most orthologous genes are conserved in the immune system, but several hundreds of genes showed clearly divergent expression between human and mouse [12]. In our previous study, co-expression and regulatory networks of hematopoietic genes were well conserved between human and mouse. The co-expression network showed “small-world” and “scale-free” architectures. The gene regulatory network formed a hierarchical structure and hematopoiesis transcription factors localized to the hierarchy’s middle level, and, also, tended to participate in blood-related diseases [13]. While scRNA-seq has been extensively applied to investigate hematopoiesis in human and mouse, cross-species comparisons of hematopoietic hierarchy have not been intensively reported, or appear only as one component of larger reports, and are relatively superficial. For example, in an early study, lineage transcriptomic characteristics were shown to be similar, without description of differentiation trajectories and consideration of transcription networks [11].

In the present work, we utilized the 10× single cell platform and a canonical correlation analysis (CCA) computational strategy, and we conducted a comparative transcriptomic analysis of the hematopoietic hierarchy in human and mouse. We found that the hematopoietic stem and progenitor cell (HSPC) compartments in the two species are composed of populations characterized by the same sets of homologous genes, and the hematopoietic lineages and transcriptional profiling in hematopoiesis are well conserved between human and mouse. Lineage specificity and maturation were mainly determined by transcription factors and their target genes. We developed a comprehensive atlas for cell type-specific regulatory sequence motifs and TF-centered regulatory networks (regulons). The primary sequence motifs of most cell type-specific TFs and their target genes were conserved between human and mouse. Our results indicate evolutionary similarity in the human and mouse hematopoietic systems.

## 2. Materials and Methods

**Figure 1:**
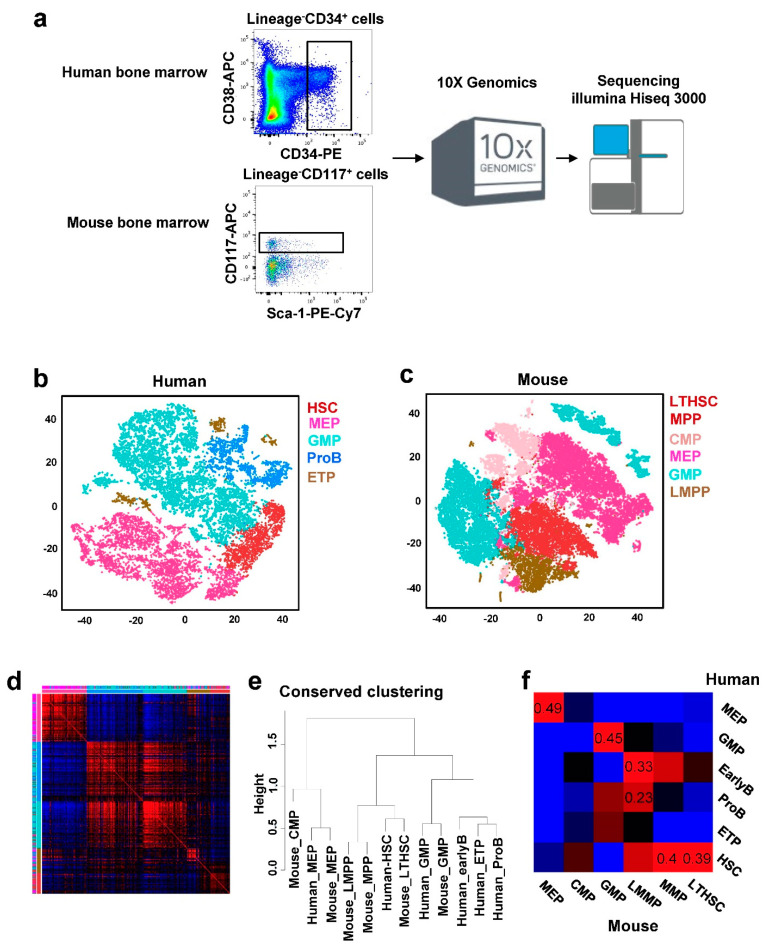
(**a**) Schematic overview of the study design. (**b**) A tSNE plot of single-cell gene expression of human HSPCs. (**c**) A tSNE plot of single-cell gene expression of mouse HSPCs. (**d**) Correlation of expression between human and mouse orthologous cell-type specific genes (red indicates high correlation and blue indicates low correlation). Cell types of species (inner was for human) were marked by the colors defined in (**b**). Conservation of homologous genes between species was evident. (**e**) The phylogenetic tree of human and mouse cell populations. Average expression across human–mouse homologous genes was calculated for cell types of human and mouse, and the distance between expression patterns for different cell types was used for hierarchical clustering. (**f**) Correlation of expression levels of homologous genes in human and mouse cell populations.

Bone marrow samples were obtained from healthy donors after written informed consent, in accordance with the Declaration of Helsinki, and following protocols approved by the National Heart, Lung, and Blood Institute (NCT00001620). CD3^−^CD14^−^CD19^−^CD34^+^ cells were sorted using a LSRII Fortessa Cytometer (BD Biosciences, San Jose, CA, USA). Lineage^−^CD117^+^ cells were sorted from bone marrow of C57BL/6 mice (Figure 1a). The Chromium Single Cell 3′ platform (10× Genomics) was used to prepare scRNA-seq cDNA libraries [14,15]. RNA-seq libraries were sequenced with paired-end reads of 75-bp on Illumina HiSeq 3000 System. The cellranger pipeline (https://support.10xgenomics.com/single-cellgene-expression/software/pipelines/latest/what-is-cell-ranger, accessed on 18 October 2018) was used to process raw data, align reads to the genome, and generate gene–cell expression matrices. Specifically, sequencing reads of human were aligned to the hg19 reference genome by STAR and uniquely aligned reads were calculated to quantitate gene expression levels for all ENSEMBL genes with unique molecular identifiers (UMIs). Low-quality cells were filtered and removed from further analyses if the number of detected genes was fewer than 500 (due to low quality, potential fragments) or more than 3000 (due to potential doublets). Cells with a high percentage of mitochondrion gene reads (>10%) were also excluded. Raw and processed data from all experiments were deposited in the NCBI Gene Expression Omnibus with GSE135194 and GSE142235 [14,15]. Downstream analyses were performed using the R software package Seurat (http://satijalab.org/seurat/, v2.3.4, accessed on 18 June 2018). Raw reads in each cell were first scaled by a library size to 10,000 and then log-transformed. Highly variable genes (~1300, identified with y.cutoff = 0.5) were used for Principal Component Analysis (PCA) of high-dimensional data. Top 30 principal components were selected for unsupervised clustering of cells with a graph-based clustering approach. Graph-based clusters methods were applied to group cells based on two-dimensional *t*-distributed Stochastic Neighbor Embedding (tSNE) using Seurat at resolution 2 [16]. Each gene from a cluster was compared to the median expression of the same gene from cells in all other clusters by FindMarkers function in Seurat and genes with *p* < 0.01 were defined as cluster-specific genes [17]. Genes then were ranked based on their expression fold change, and top cluster-specific genes were compared with published cell type-specific genes [1,2]. An HSPC subtype was assigned to each cluster based on statistical significance of overlap between HSPC- and cluster-specific genes (with smallest *p* values of Fisher’s exact test) [17]. Sequencing reads of mouse were aligned to the mm9 reference genome by STAR and the gene–cell expression matrix was calculated. Subsequently, gene expression analysis for mouse followed the same pipeline as for human, using cell lineage specific genes derived from GSE81682 in GEO as references for cell type assignment.

We used the canonical correlation analysis (CCA) algorithm to perform comparative transcriptomic analysis of the hematopoietic system between human and mouse (RunCCA function in Seurat 2 with parameter of num.cc = 20). The CCA algorithm is a multivariate statistical technique for the determination of linear associations between two sets of variables that are maximally correlated. In scRNA-seq analysis, the CCA algorithm can detect the statistical common factors between two digital gene expression (DGE) matrices, which vary due to batch effects or different methods used in normalization procedures. These factors are aggregations of conserved gene-to-gene correlations between human and mouse, and, therefore, we could align all human cells against all mouse cells in an identical linear space and visualize shared populations in different species with further analyses such as t-SNE.

To explore the conservation of cell populations between human and mouse, we applied scmap (http://bioconductor.org/packages/scmap, accessed on 10 July 2019) to project cells from a human scRNA-seq dataset onto cell types defined in the mouse scRNA-seq dataset, with a parameter threshold of 0.7, and, conversely, from human to mouse datasets. Cells in human were expected to be projected to the closest mouse cell types if there was transcriptional conservation.

Differentiation trajectory analyses were conducted with Monocle (https://www.bioconductor.org/packages/release/bioc/html/monocle.html, accessed on 6 June 2019). Preprocessed Seurat objects were imported into Monocle with the “importCDS” function. Monocle’s “orderCells” function arranged cells along a pseudo-time axis to indicate their position in a developmental continuum. The trajectory trees identified by Monocle were colored by cell types or expression levels of marker genes to show the differentiation directions during hematopoiesis. The reversed graph embedding algorithm in Monocle 2 was used to impute differentiation trajectories in both 2- and 3-dimensions.

With RcisTarget software in Bioconductor, we identified putative transcription factor motifs; SCENIC algorithm was used to construct gene networks and model regulon activity within each cell state. We followed the developer’s instructions (https://github.com/aertslab/SCENIC, accessed on 18 May 2019) pipeline for these analyses. For each cell type-specific gene list of human and mouse, we scanned two motif TFs databases (using RcisTarget in Bioconductor on hg19-tss-centered-10 kb-7species.mc8nr.feather or mm9-tss-centered-10 kb-7species.mc9nr.feather from https://resources.aertslab.org/cistarget/, accessed on 10 April 2019) and retained modules with significant motif enrichment [18].

For regulon analysis, the expression matrices of human and mouse were first extracted from Seurat objects and then transformed into the required format for SCENIC, in which rows represented genes and columns represented cells [18]. Cells with less than 500 and more than 3000 detected genes were filtered. We also filtered genes with less than at least 6 UMI counts in human and mouse. We used GENIE3 in the Bioconductor expression matrix to identify co-expressed gene modules and infer potential TF targets for each module. Regulatory modules (regulons) were identified from co-expression and DNA motif analyses. Regulons were then evaluated in each cell to ascertain their activities by the AUCell package in Bioconductor, before a binary matrix (with 1 for active and 0 for inactive, with threshold determined by the distribution of AUCell scores) was obtained. To profile gene regulatory module features of all HSPCs, the Spearman correlation coefficients between regulons were calculated, and only the positive-correlated targets in the regulons were retained. We chose top5perTarget as a parameter of coexMethod to run SCENIC [18], such that the top 5 percent of TFs were employed to create regulons for each gene. Cell specificities of the identified regulons were assigned with Fisher’s exact test using the mark gene list [1,2]. topGO was used to functionally annotate the identified regulons [19]. Networks of the TF regulons with motif information were visualized by Cytoscape [20].

## 3. Results

### 3.1. scRNA-Seq Identified a Comprehensive and Conserved List of HSPC Types

We obtained bone marrow samples from four healthy human donors. In order to characterize the early stages of hematopoiesis, we sorted lineage^−^CD34^+^ cells to enrich for HSPCs. After filtering out cells with limited numbers of detected genes, our dataset contained 15,245 single CD34^+^ stem/progenitor cells. Sequencing data of single CD34^+^ cells, as visualized tSNE (Figure 1b), displayed clear clusters, suggesting distinct cell types at the molecular level. Hematopoietic cell identity was assigned to each cell cluster by comparing cluster-specific genes with a reported lineage signature gene list [1,2]. CD34^+^ cells were clustered into 15 clusters and then could be computationally assigned to the following cell populations: multipotent progenitor HSCs, megakaryocyte–erythroid progenitors (MEPs), granulocyte–monocyte progenitors (GMPs), B lymphocyte progenitors (ProBs), and early T lineage progenitors (ETPs) (Figure 1b). The number of clusters identified by Seurat depends on the resolution selected. Although resolutions 1, 2 and 3 generated different numbers of clusters, cell type assignment at each resolution was almost identical and did not affect further analyses.

For analysis of murine hematopoiesis, 17,560 linage^−^CD117^+^ cells from B6 mice were also clustered, unsupervised, based on transcriptome similarity using tSNE (Figure 1c). Hematopoietic cell identity was assigned to each cluster of cells by comparing cluster-specific genes with accepted lineage signatures. We could group the cells into 36 clusters and then assign them into long-term hematopoietic stem cells (LTHSC), multipotent progenitors (MPP), lymphoid multipotent progenitors (LMPP), common myeloid progenitors (CMP), MEP, and GMP.

We defined HSC in human and MPP and LTHSC in mouse as conserved HSC; ProB in human and LMPP in mouse as lymphoid cells; and GMP and MEP in both species as GMP and MEP, respectively. We considered that these were species comparable/closest cell populations [21,22].

**Figure 2:**
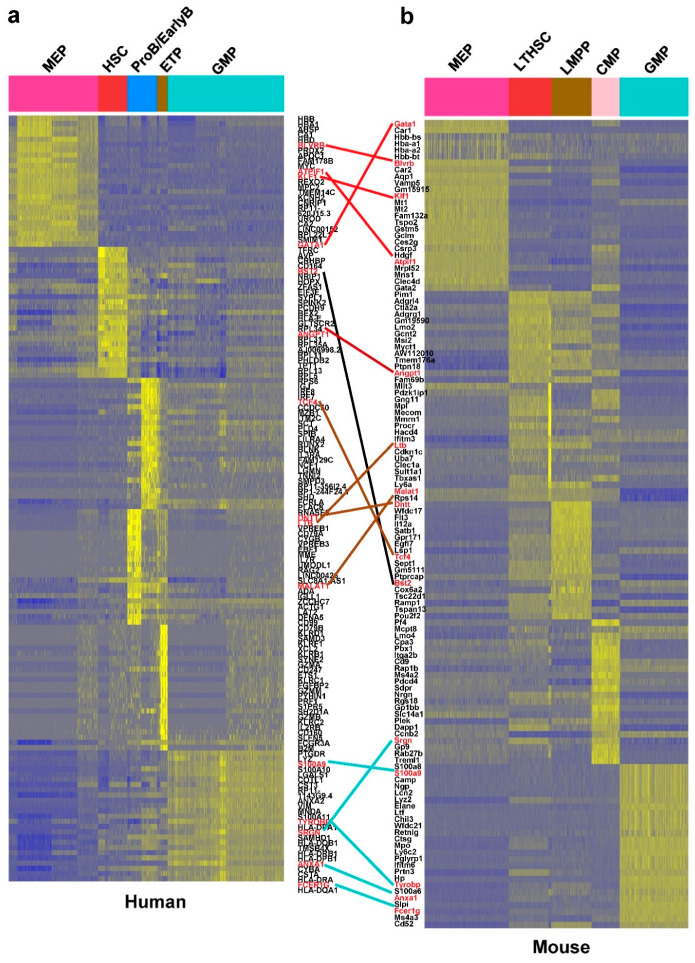
Heatmaps of differentially expressed genes in (**a**) human and (**b**) mouse cell populations. Heatmaps showed a scaled gene expression of the top 25 genes representing each of the identified cell populations found in human and mouse. Each row represents one gene and each column displays gene expression of 5 pooled cells. Genes are listed in the middle and homologous genes were linked together (colored by the cell type specificity). The marker gene lists of concordant cell populations in human and mouse tend to be homologous. Note that MEP and GMP were well characterized, but others were not well characterized in human and mouse due to the characteristic of cell populations.

We analyzed human and mouse datasets in parallel with orthologous genes in InParanoid [23]. tSNE plots showed cells grouped by species, instead of by a cell type, due to species specificity and batch effects (Figure S1a, human and mouse cells were profiled at different times). The CCA algorithm is a multivariate statistical technique for finding linear associations between two sets of variables that are maximally correlated. In scRNA-seq analysis, the CCA algorithm can detect the statistical common factors among two digital gene expression (DGE) matrices, which vary from each other due to batch effects or different methods used in normalization procedures. After alignment with CCA, cells of mouse and human were well mixed and separated into the same cell type categories (Figure S1b,c). The cells clustered into 17 subpopulations (15,245, 17,560 cells, and 17, 16 subpopulations contain cells from human and mouse, respectively) by computational analysis (Figure S1c). Cell assignments were validated from expression of typical cell type-specific genes (Figure S1d). Complete gene lists in different cell populations were shown in Supplemental File 1. There were many homologous genes apparent in the two gene lists for the same cell types of human and mouse. The top 25 specific genes and their expression are shown in Figure 2, in which the homologous genes were highlighted and linked with lines. Typical hematopoiesis-related genes and their expression in different cell populations are shown in Figure S2.

### 3.2. Conserved Cell-Type Expression between Human and Mouse

We built a human-to-mouse one-to-one orthologous gene list (13,520 genes), collected from InParanoid (http://inparanoid.sbc.su.se, accessed on 16 August 2018) for homology analysis [23]. Matching cell types requires shared expression patterns between species, and we found that there were many homologous genes that best discriminated mouse and human HSPCs. Identification of homologous types or classes enabled an analysis of conservation and divergence of gene-expression patterns between the two species. For each pair of homologous types, we compared the expression of orthologous genes and investigated cell type-specific genes between human and mouse. There were a high number of genes in human and mouse which shared the same cell-type specificity (Rand index = 0.24, *p* < 0.00001, Supplemental File 1), especially in MEP and HSC. There were many divergent genes that only express in certain lineages of human, not in those of mouse, and vice versa (Supplemental File 1). We collected the overlap of type-specific gene lists in human and mouse, calculated averages of gene-to-gene correlations with human and mouse datasets, and conducted a clustering analysis with the averaged correlations (Figure 1d). The column annotations for the assigned cell types of marker genes in human (inner) and mouse (outer) showed high consistency, implying a conservation of cell type-specific genes.

We explored similarities and differences in HSPCs between two species. To derive a quantitative view of the cellular evolution from mouse to human in hematopoiesis, we used mouse and human orthologous genes and calculated an average of expression of cells of each type for human and mouse. After hierarchical clustering (ward.D2 method, with 1-Pearson correlation as distance), a dendrogram indicated that cell types were highly conserved between the two species (Figure 1e). For example, the MEP of mouse and human shared very similar transcriptomes, and a GMP also showed high similarity. Human HSC were first clustered with mouse LTHSC by the nearest neighbor (Figure 1e), and then with mouse MPP. MPP and LMPP in mouse had a similar transcriptome, which was already observed in human [1]. The high correlations of human and mouse in the same cell types showed that cell-type similarity in orthologous gene expression dominated species differences, particularly for the MEP and HSC populations (Figure 1e), as observed by others [8,18,24].

A heatmap in Figure 1f quantitatively presents the expression similarity of cell types in human and mouse. The mouse MEP cluster shows strong correlations with the human MEP (R = 0.496, *p* < 1 × 10^−10^), and the human HSC shows strong correlation with mouse LTHSC (R = 0.389, *p* < 1 × 10^−10^) and MPP (R = 0.395, *p* < 1 × 10^−10^). Conservation of mammalian cell types in single-cell comparative genomic studies have been reported [11,24].

### 3.3. Projection by Scmap and Conservation of Cell Populations between Human and Mouse

**Figure 3:**
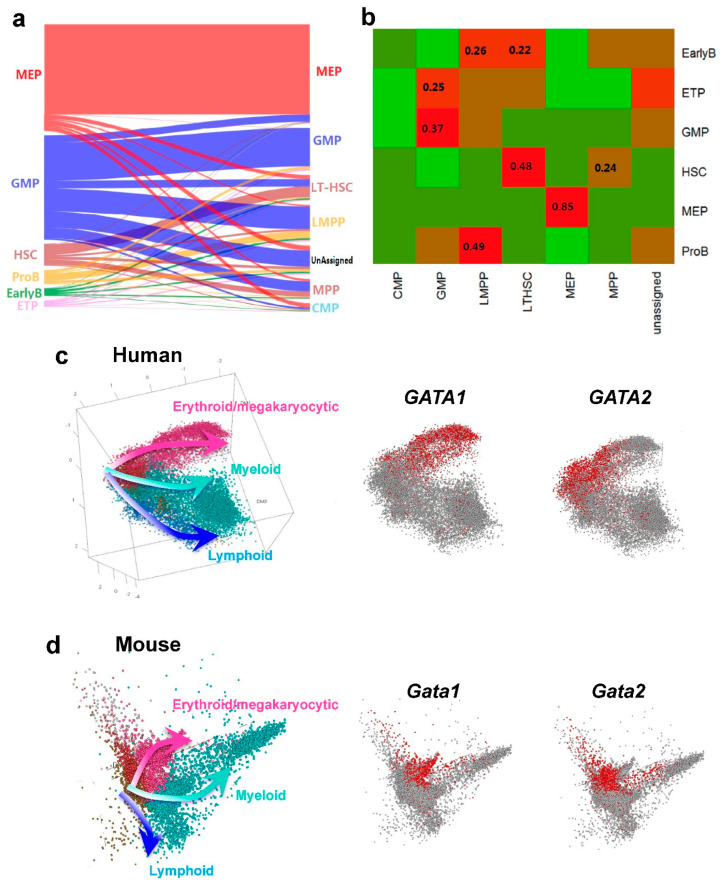
(**a**) Sankey diagrams of scmap-cluster projection of human dataset to mouse dataset. Each row is a cell of human, colored with cell types. (**b**) Fractions of human cell populations projected to mouse cell types. Cells tended to be projected to the corresponding cell population defined in mouse; for example, 85% of MEP mouse cells were mapped to human MEP cell types. (**c**,**d**) Monocle revealed linage differentiation trajectories of human (**c**) and mouse (**d**) HSPCs. Cells (balls, colored based on predicted cell type) are arranged in a 2D space calculated with Independent Component Analysis. The results obtained with Monocle 2 are shown in Figure S3, showing the same themes. Expression of *GATA1* and *GATA2* for lineages are highlighted in a differentiation tree, showing a clear *GATA2* and *GATA1* switch during differentiation.

The sharing of marker genes shown in Supplemental File 1 and Figure 2 simply confirmed the comparable cell populations of human and mouse. To be more quantitative, single-cell transcriptomes of human cells were compared with those of mouse cells using scmap [25]. scmap projects a cell onto a reference dataset, allowing inference of cellular identity based on resemblance of transcriptomes to the reference cell type. Seurat was used to normalize gene expression by library sizes and log2 transformed the data, and libraries were subset by expression of shared genes across datasets. Most human MEP cells (85%) were mapped to mouse MEP cell types based on transcriptional similarity, suggesting functional similarity and species conservation. A total of 48% of human HSC cells were mapped to mouse LTHSC cell types, and 24% were mapped to MPP, indicating the similarity of MPP and HSC (Figure 3a,b). Other human cell types were also mainly mapped to their closest murine cell types. The mapping results are consistent with known human–mouse comparable/closest cell populations [21,22]. In total, our analysis confirmed conservation of hematopoietic stem and progenitor cell types between human and mouse.

### 3.4. Developmental Trajectories in Human and Mouse Hematopoiesis

Clustering is based on an assumption of biologically distinct groups, such as discrete cell types or states; pseudo-temporal ordering assumes that data lie on a connected manifold [26]. For detailed analysis of the transition from stem cells to lineage-restricted progenitors, we used Monocle to arrange each cell by pseudo-temporal ordering based on gene expression [27]. After applying Monocle to the profiled human and mouse cells, an intuitive graphical representation of early stages of HSPC differentiation emerged. In human, lineages clearly separated among lineage^−^CD34^+^CD38^+^ progenitors (Figure 3c). We defined HSC in human and LTHSC in mouse as roots, so that they were located at starting points of the hierarchy. In both human and mouse, three branches arose from HSC and LTHSC. We confirmed cells in the three branches as erythroid/megakaryocytic, myeloid and lymphoid (Figure 3c,d). At the cellular level, the adjacency of cell types on plotting reflects differentiation pathways. The differentiation trajectories of human and mouse are highly similar, as described by others [1,2]. The newly defined model shows unexpected developmental shifts within the progenitor cell architecture: where many stem and progenitor cell types are multipotent, the stem cell compartment is multipotent and only progenitors are unipotent. These features do not present in the classic hierarchical model. We also examined expression level changes of individual genes within the trajectories. As an example, *GATA1* expression increased along the erythroid branch, and *GATA2* expression decreased with differentiation. There was a *GATA1* and *GATA2* switch in human and mouse, showing species conservation of gene participations during erythroid differentiation (Figure 3c,d) [28]. Expression changes of other representative lineage-specific genes along human and mouse differentiation trajectories also were shown in Figure 3c,d and Figure S3. Therefore, at both cellular and molecular/gene levels, human and mouse show conservation during differentiation. scRNA-seq allowed for deconvolution of heterogenous HSPC population, both LTHSC and CD34^−^ as stem cells and lineage-committed progenitors, and for the reconstruction of a trajectory of normal hematopoietic differentiation in human and mouse. Monocle 2 was also used to analyze the data and produce 2- and 3-dimensional projections, which were similar to those generated with the Monocle ICA algorithm (Figure S4) [29]. Conservation of hematopoietic differentiation between human and mouse was evident from the observations that genes were activated at the same differentiation stages across species [9], and both human and mouse cells were distributed along pseudo-temporally ordered paths from HSCs/LTHSCs to three branches—erythroid/megakaryocytic, myeloid, and lymphoid.

### 3.5. Conserved Cell Type Specific Regulatory Elements/Motifs between Human and Mouse

**Figure 4:**
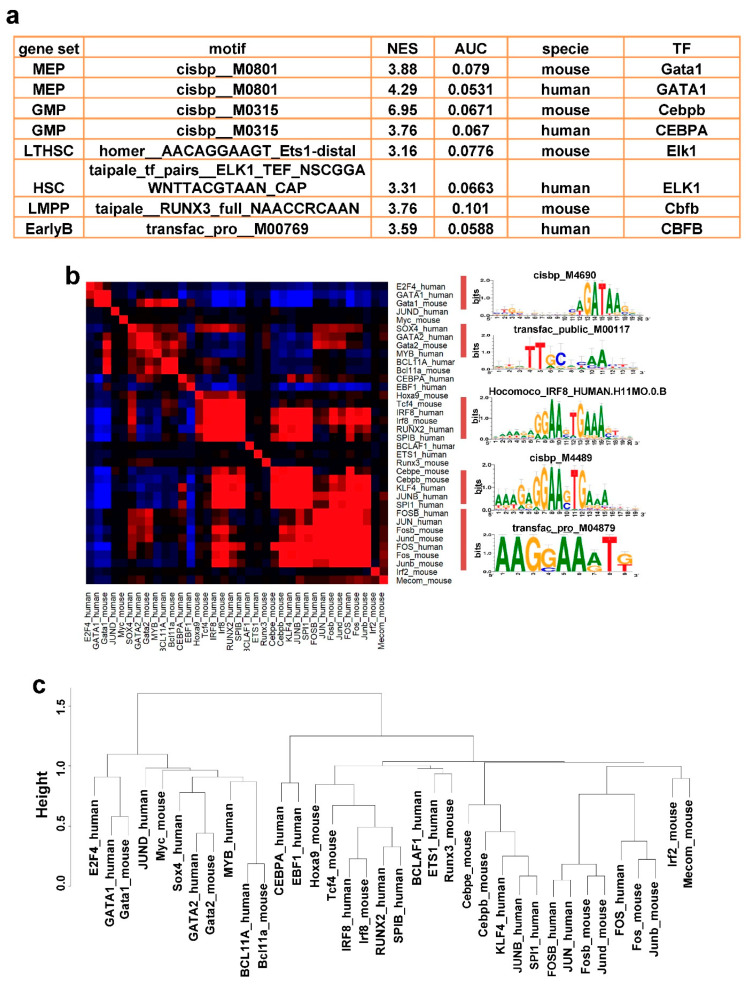
(**a**) A table of enriched motifs by RcisTarget for selected sets of regulons related to cell populations in human and mouse, generated within the SCENIC workflow. (**b**) Average AUCell scores of all human and mouse cells were calculated with the identified regulons to obtain a regulon-cell matrix. Then, the distance among regulons was hierarchically clustered. The corresponding binding motifs were shown on the right. Regulons were grouped into several major modules, along with representative TF regulons and associated cell types. (**c**) A clustering dendrogram shows the relationships of 37 orthologous TF regulons.

Given a gene list, the program RcisTarget identifies over-represented TF-binding motifs and can predict candidate target genes (regulons) based on databases containing motifs with genome-wide rankings. The over-representation of each motif for the gene list is estimated through calculating a normalized enrichment score (NES) by AUCell algorithm. After the cell type-specific gene lists were input for calculation, 1494 (in human) and 663 (in mouse) TFs with their recognition motifs were identified as significantly enriched in different cell populations. We found that there were species conservation of transcription factors and recognition motifs (Figure 4a) in the most similar cell types. As shown in Figure 4a, MEP gene-set was enriched for a *GATA1* associated “cisbp__M0801” motif (NES = 4.29), and this motif was also enriched in mouse with NES = 3.88. GMP gene-set was enriched for a *CEBPA* associated “cisbp__M0315” motif (NES = 6.95), and this motif was also enriched in mouse with NES = 3.76.

The complete list of well-defined cell type-specific motifs, their corresponding transcription factors for human and mouse, as well as species conservation, are shown in Supplementary File 2. Some motifs showed species conservation and were active in only one cell population. Some motifs were species-specific, restricted in their appearance, only among human or mouse cell populations. Hematopoietic differentiation is controlled by key transcription factors (TFs), which regulate stem cell functions and differentiation. The same TFs in human and mouse tend to contribute to the same hematopoietic lineage differentiation, but some TFs only function in hematopoiesis of human or mouse. For example, in both human and mouse, higher activities of *GATA1*- and *E2F1*-related motifs were present in MEP cells; *SRF* and *RELA* motifs in GMP cells; *IRF* and *CBFB* motifs in lymphoid cells; and *GATA2* and *CHD1* motifs in HSC cells were observed. In addition, other TFs were identified in specific cell populations of both species, such as upregulation of *FOSB*, *JUN*, and *JUND* in HSC cells, and higher activity of *JUN*, *JUNB*, *GTF2B* and *CEBPD* in lymphoid cells. Antagonism between transcription factor *PU.1* (encoded by *SPI1*) and *GATA2* drives myeloid/lymphoid versus erythroid/megakaryocyte lineage commitments [30,31]. In both human and mouse, *GATA1* is the key transcriptional factor for erythro- and megakaryocytic differentiation; *GATA2* downregulation and reciprocal *GATA1* upregulation maintain cell differentiation in the erythroid/megakaryocytic lineage [30,31,32]. We observed higher activities of *GATA1* motifs in MEP, while *SPI1* motifs were exclusive in GMP cells of human and mouse. Transcription factor *MEIS1* is an HSC marker, and its expression level declines with cell differentiation; it is known to promote expression of stem cell markers in leukemias [33,34]. We observed *MEIS1* motifs exclusively activate in LTHSC and MPP of mouse, indicating higher activity in stem cells and early stage of multipotent progenitors. The cell type expression specificity of motifs is consistent with function. *FOSB*, *JUN*, *JUND* and *JUNB* are members of activator protein-1 (AP-1). AP-1 is involved in cell differentiation, proliferation and survival [35]. *JUNB* is required for Th17 cell development [36]. The expression of *FOSB*, together with *GFI1*, *RUNX1* and *SPI1*, are sufficient to generate immunocompetent HSCs in adult mouse endothelial cells [37]. Progression from the CLP to B/Myeloid and ProB Precursors during B Lymphopoiesis requires *CEBP*. *CEBPA* is required for Flt3^+^ CLP maturation into ProB cells and then for proliferation [38]. Collectively, multiple TFs, alone and in combination, regulate hematopoietic stemness and differentiation.

### 3.6. Conserved Cell Type Specific Regulatory Networks between Human and Mouse

To recapitulate the gene regulatory relationships among the different populations, and thus infer the regulatory mechanisms underlying hematopoiesis, we used SCENIC, an algorithm to deduce Regulatory Networks and cellular status from scRNA data [18]. A cell expression matrix of 10,000 highly variable genes extracted from Seurat object (data slot after normalization) was imported as an input matrix for SCENIC. By identifying the co-expression modules (including transcriptional factors) and analysis of cis-regulatory motif analysis with each co-expression modules, we obtained 84 cell identity-specific regulons, together with their corresponding targets (Supplementary File 3). The number of targeted genes ranged from 4 to 587 (average, 63).

Next, we investigated the gene networks that underlie mammalian cell-type conservation. For example, the GATA1 regulons for MEP in human and GATA1 regulons for MEP in mouse were both identified as enriched regulons (Figure 4b,c). We examined TF regulons from both human and mouse in SCENIC and identified 24 orthologous TF regulons, which could be grouped into five major modules across human and mouse (Figure 4b and Supplementary File 3). As examples, *GATA1* and *E2F4* are associated with erythropoiesis, and *IRF8* is associated with GMP populations. These modules were enriched not only for lineage-specific transcription factors but also for conserved binding motifs that lead to coordinated module activation in lineage commitment (Figure 4b, right). In contrast to a published work [24], we did not find much species-specific genetic regulation between human and mouse. We also clustered regulons based on a Pearson correlation of their AUCell scores in all cells (Figure 4c), and expanded to include extended motifs defined in SCENIC (Figure S5c). Again, clustering was driven by cell types rather than by species. This reflected that homologous genes played dominant roles in the clustering results. If non-homologous genes expressed in only human or mouse contribute heavily to variation, regulons would be clustered into two groups (human and mouse) rather than being grouped by the same/similar regulons across the two species.

We used the Fisher’s exact test to check the cell type specificity of regulons, and the result is shown in Supplementary File 4. Briefly, to test the enrichment of a cell type annotation for a regulons, we used a One-tailed Fisher’s exact test to determine the significance of the association between gene members in regulons and marker gene list, and thus determined the enrichment of cell type for the regulons [17]. Most regulons are cell type-specific, and the regulons associated with same transcriptional factors were assigned to the same cell populations in human and mouse. For example, *GATA1* regulons were assigned to the MEP of human, and the MEP and CMP of mouse. *IRF* regulons were assigned to the GMP of human and mouse. Some regulons were not cell type-specific, such as *SP1* and *JUND*. These regulons were active in many cell populations, indicating their importance in the whole stage of differentiation.

We also examined the potential regulatory role of novel transcription factors by a gene ontology (GO) enrichment analysis of target genes in 23 novel regulons. Functional annotations of regulons are provided in Supplementary File 5. Enriched GO terms for *GATA1* are associated with the activation/differentiation of erythropoiesis terms GO:0030218 (erythrocyte differentiation) and GO:0034101 (erythrocyte homeostasis). *YBX1* regulon was annotated with GO:0030218 (erythrocyte differentiation); *YBX1* is a transcription factor widely expressed in all cellular lineages during differentiation and is involved in erythroid cell development [39]. Under regulation by *GATA* factors, *YBX1* functions in erythroid differentiation and aberrant expression of *YBX1* gene results in dyserythropoiesis [39,40]. *IRF8* targeted genes were highly related to biological processes, such as immune response and lymphocyte activation, with terms of GO:0006955 (immune response), GO:0001775 (cell activation), and GO:0030217 (T cell differentiation).

After more detailed exploration of the same-TF regulatory networks inferred for regulons between human and mouse, we found that there was a significantly higher number of conserved targets (evaluated by the Fisher’s test for significance of overlap), and some species-specific targets genes, as reported by another study [18]. Two examples of such an analysis are shown in Figure S5a,b. For regulons generated with SCENIC using the target genes of *IRF8* in human and mouse, the edges represent the connections between each of the two TFs and their target genes. Human and mouse shared 34 targeted genes, significantly higher than predicted by chance (*p* < 1 × 10^−60^, Fisher’s test). For regulons generated with SCENIC using the targeted genes of *GATA1* in human and mouse, there were seven shared targeted genes (*p* < 1 × 10^−10^, Fisher’s test). Species conservation of hematopoiesis was apparent at the gene network level, both for TFs and their targets.

**Figure 5:**
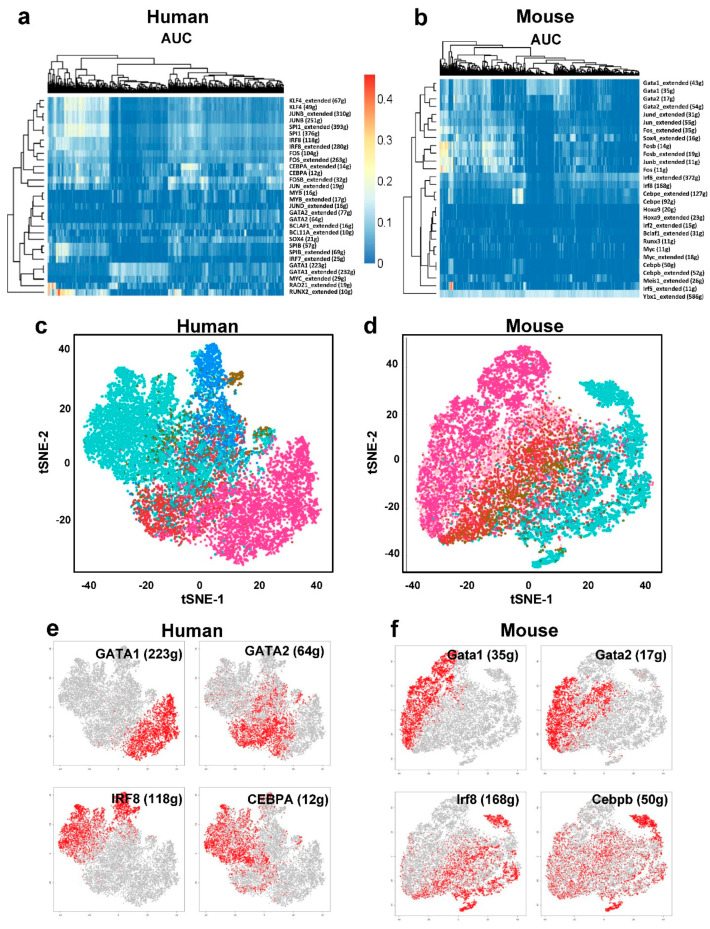
(**a**) A heatmap of the AUCell scores for the activities of regulons in each cell of human; the regulators for same cell populations were clustered together. Right are transcription factor and number of target genes. (**b**) A heatmap of the AUCell scores for the activities of regulons in each cell of mouse, the regulators for same cell populations were clustered together. (**c**) Binary regulon activity scores based tSNE plots for human (t-SNE was run with the binary regulon activity matrix as input). Each dot is a cell, which is colored by cell types. (**d**) Binary regulon activity scores based tSNE plots for mouse. Each dot is a cell, which is colored by cell types. (**e**,**f**) Same as (**c**,**d**), but cells are colored by AUCell scores of regulons, with red meaning high AUCell scores.

SCENIC uses the AUCell algorithm to score the activities of entire gene regulatory networks or regulons in each cell, which can be clustered and displayed in activity matrices (Figure 5a,b). To determine the “on/off” activity of each regulon, AUCell automatically identifies the threshold of activity and generates the binary activity regulon matrix (1 for active, 0 for inactive). Next, the t-SNE algorithm was used to project all cells onto the two dimensions, based on the binary regulon activity matrix, to examine whether it can accurately identify cellular types. Consistent with the Seurat tSNE algorithm, cell clustering using regulon activity clearly distinguished different cell populations (Figure 5c,d), confirming the characterization of cell identity by tSNE based on the activities of regulons by integrating the expression of TFs and their targets. Cell clustering using activities of regulons, rather than by gene expression, resulted in t-SNE projections to distinct cell populations. The activation of cell-type-specific networks was conserved between human and mouse (Figure 5e,f), which was evident from conserved regulon activities for human and mouse in the same cell populations.

## 4. Discussion

Our comparative transcriptomic analysis of the hematopoietic system revealed evolutionary conservation in the hematopoietic hierarchy across human and mouse. We found that HSPC compartments in the two species were composed of populations characterized by lineage-specific regulators. The lineage differentiation patterns and transcriptional profiling were well conserved between human and mouse, indicating evolutionary similarity in their hematopoietic systems. Further, we examined the TF activities that may contribute in maintaining differentiation during hematopoiesis and linked those to target genes [41]. A set of TF regulons, defined by TF-to-target correlation and TF motif analysis, was identified for different cell populations, and the regulons of human and mouse are highly conserved.

In comparing human and murine transcriptomes in hematopoiesis, one challenge is a lack of appropriate, species-matched reference gene lists, which are needed to assign cell type; the complicated hierarchical relationships between cell lineages exacerbates this problem [42]. Although characterization of the transcriptional status of individual cells enables imputation of differentiation trajectories, scRNA-seq measurements are limited by large fractions of dropouts. A new algorithm to impute dropout, and technology to increase coverage, help to improve the accuracy of trajectory inference. the integration of different types of data is a further advance. A recent study applied scRNA-seq and scATAC-seq data from bone marrow to gain deeper insight into differentiation trajectories [43]. Species conservation of a differential trajectory should be better elucidated when scRNA-seq and scATAC-seq data are available for both human and mouse.

We used regulon’s activities to elucidate cellular differentiation lineages, and the result supports computational approaches to analyzing gene activation in comparing species. There is a smaller batch effect in TF regulon activation than in gene expression in single cell data, so that conclusions concerning network activity are robust and can be exploited to overcome batch or technical effects [18]; further, cell alignment is unnecessary, which is challenging due to computational complexity when integrating mega-scale single cell datasets in the future [16]. A gene regulation-based approach is a good complement to expression analysis on single cell data.

When we calculated motif enrichment and estimated regulon activities, gene regulation showed lineage conservation between human and mouse. scATAC-seq provides a more direct measurement of genome-wide activity of enhancers and promoters [44]. Chromatin accessibility is also evolutionarily conserved [45]. Thus, a joint measurement of gene expression and chromatin accessibility of the same cells in human and mouse will enable a deep comparison of the regulatory and transcriptomic landscape of hematopoiesis [46]. The current study only analyzed protein coding genes; inclusion of miRNA and lncRNA will add more layers of complexity to gene regulation to assess species conservation of haematopoiesis [15,47,48].

We also downloaded the datasets from GSE81682 (mouse) and The Human Cell Atlas (human), and analyzed them with comparable computational strategies [2,49]. Similar results were obtained when we compared hematopoietic transcriptome between human and mouse. Part of our results, including the cell lineage assignment and differential trajectories, are shown in Figure S6. The cells in these two datasets follow the same differentiation trajectories as those obtained with our datasets. The results of our and third-party datasets showed that the conservation of gene regulation resulted in the similarity of gene expression in human and mouse. Transcription similarity can help in guiding the exploration of human physiological and pathological hematopoiesis with mouse models [50].

## Data Availability

The datasets generated and analyzed during the current study are available in the GEO repository, with accession numbers GSE135194 and GSE142235.

## Supplementary Materials

The following are available online at https://www.mdpi.com/article/10.3390/cells10050973/s1, Figure S1: tSNE plots of HSPCs from human and mouse before and after alignment, Figure S2: Typical genes and their expression in cell populations, Figure S3: Expression of characteristic genes for lineages, Figure S4: Monocle 2 identifies major branches of human and mouse hematopoiesis, Figure S5: Shared targets between regulons from human and mouse, Figure S6: tSNE plots and trajectories from human cell atlas and GSE81682, Supplementary File 1: cell type specific genes, Supplementary File 2: regulatory motifs, sequence logos and their corresponding cell types, Supplementary File 3: identified regulons by SCENIC with gmt format, Supplementary File 4: cell type specificity of regulons, Supplementary File 5: functional annotations of regulons.
