# Supplementary figures and images for: Comparative Transcriptomic Analysis of the Hematopoietic System between Human and Mouse by Single Cell RNA Sequencing
Source: Cells. 2021 Apr 21;10(5):973. doi: 10.3390/cells10050973 (PMC8143332; doi:10.3390/cells10050973)

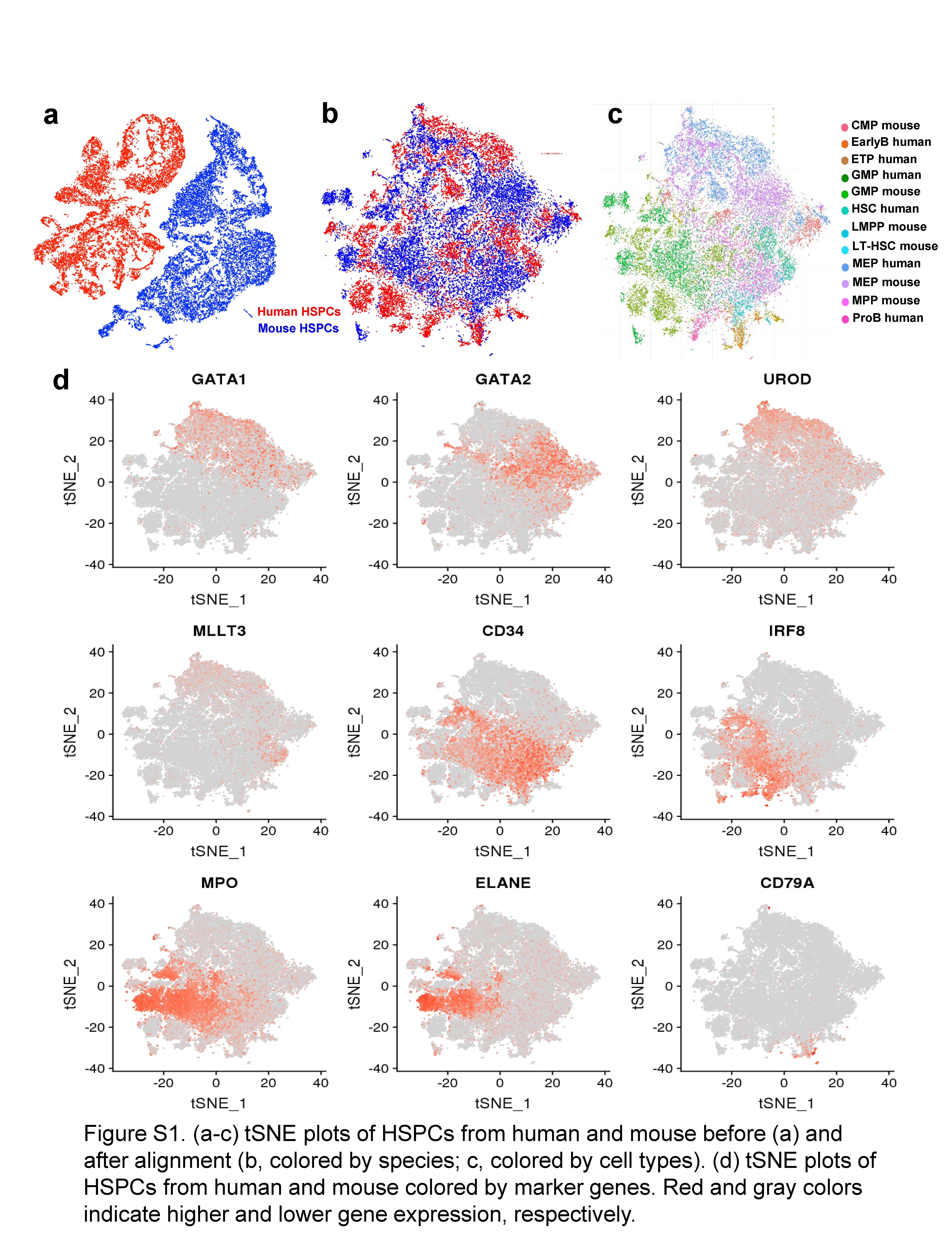

Supplement: Supplementary file 1 [file cells-10-00973-s001.zip › supplementary materials/Fig_S1.jpg]

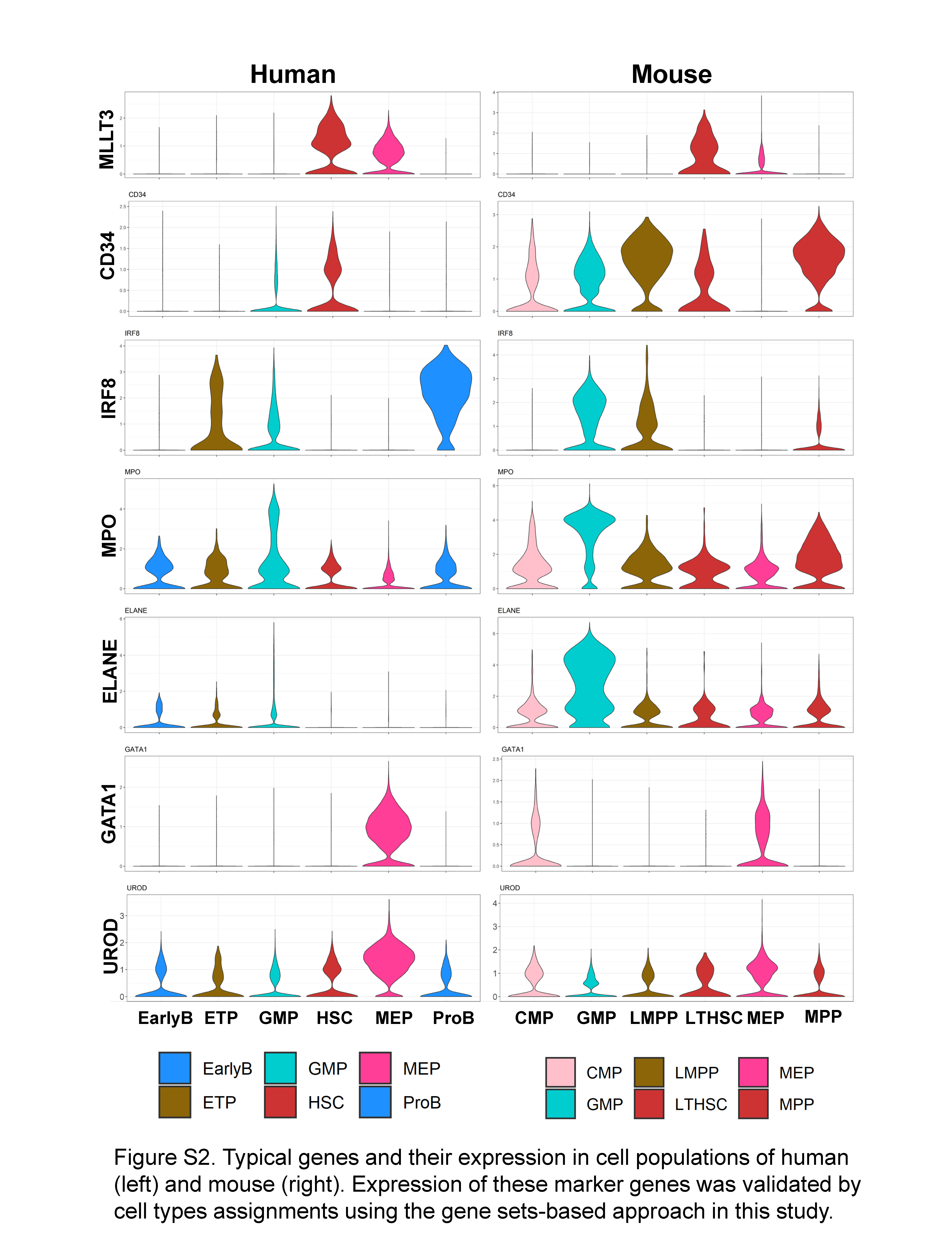

Supplement: Supplementary file 1 [file cells-10-00973-s001.zip › supplementary materials/Fig_S2.jpg]

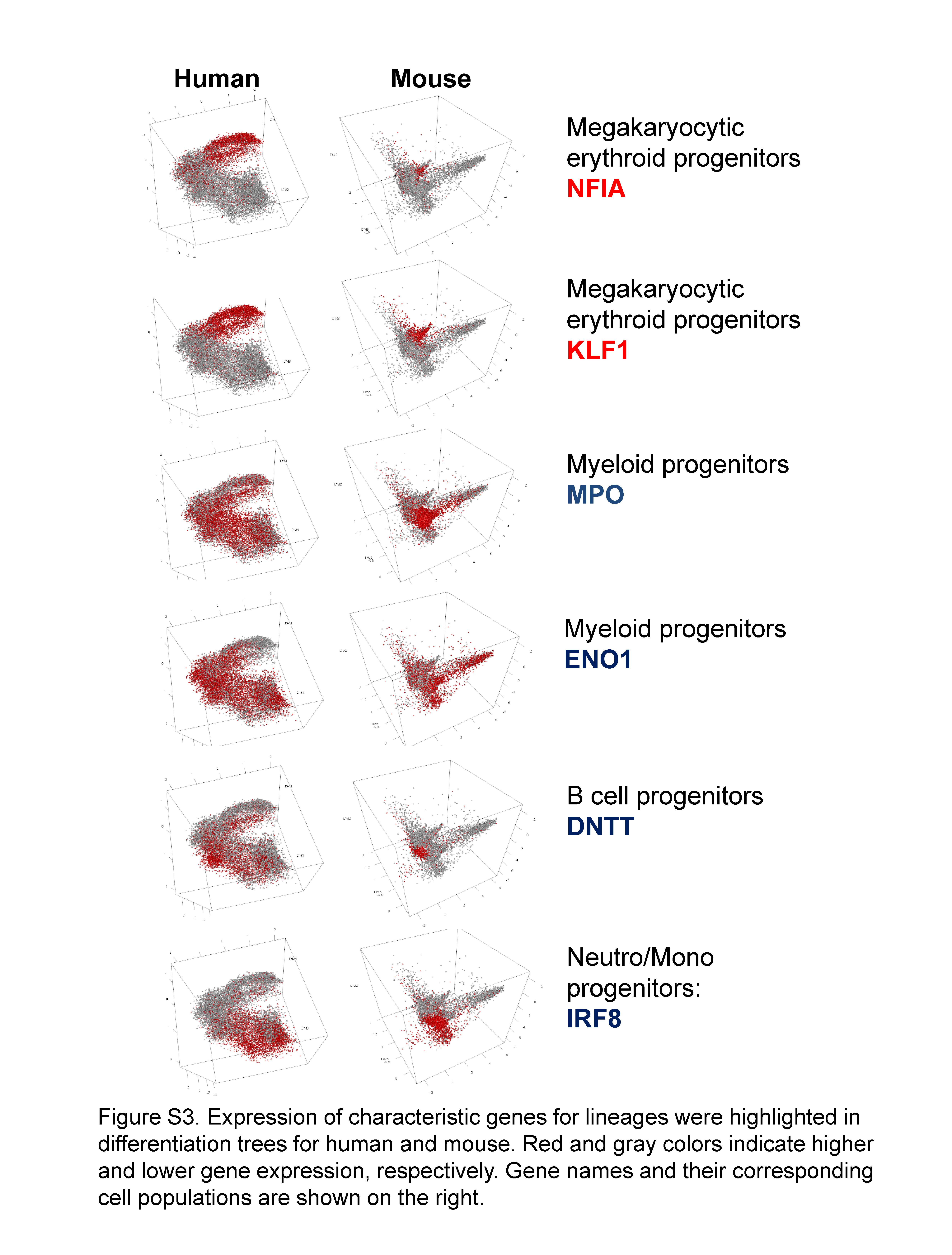

Supplement: Supplementary file 1 [file cells-10-00973-s001.zip › supplementary materials/Fig_S3.jpg]

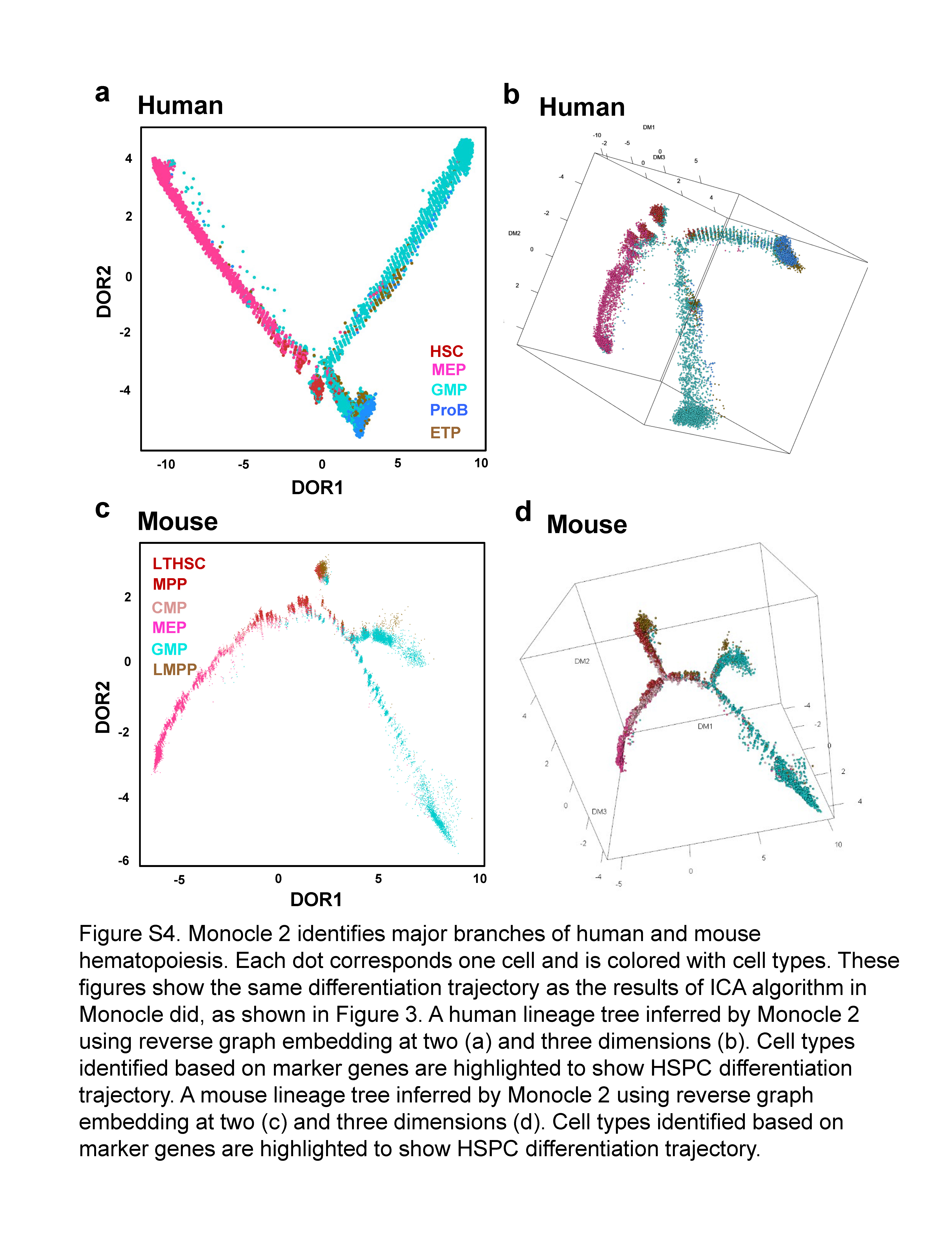

Supplement: Supplementary file 1 [file cells-10-00973-s001.zip › supplementary materials/Fig_S4.jpg]

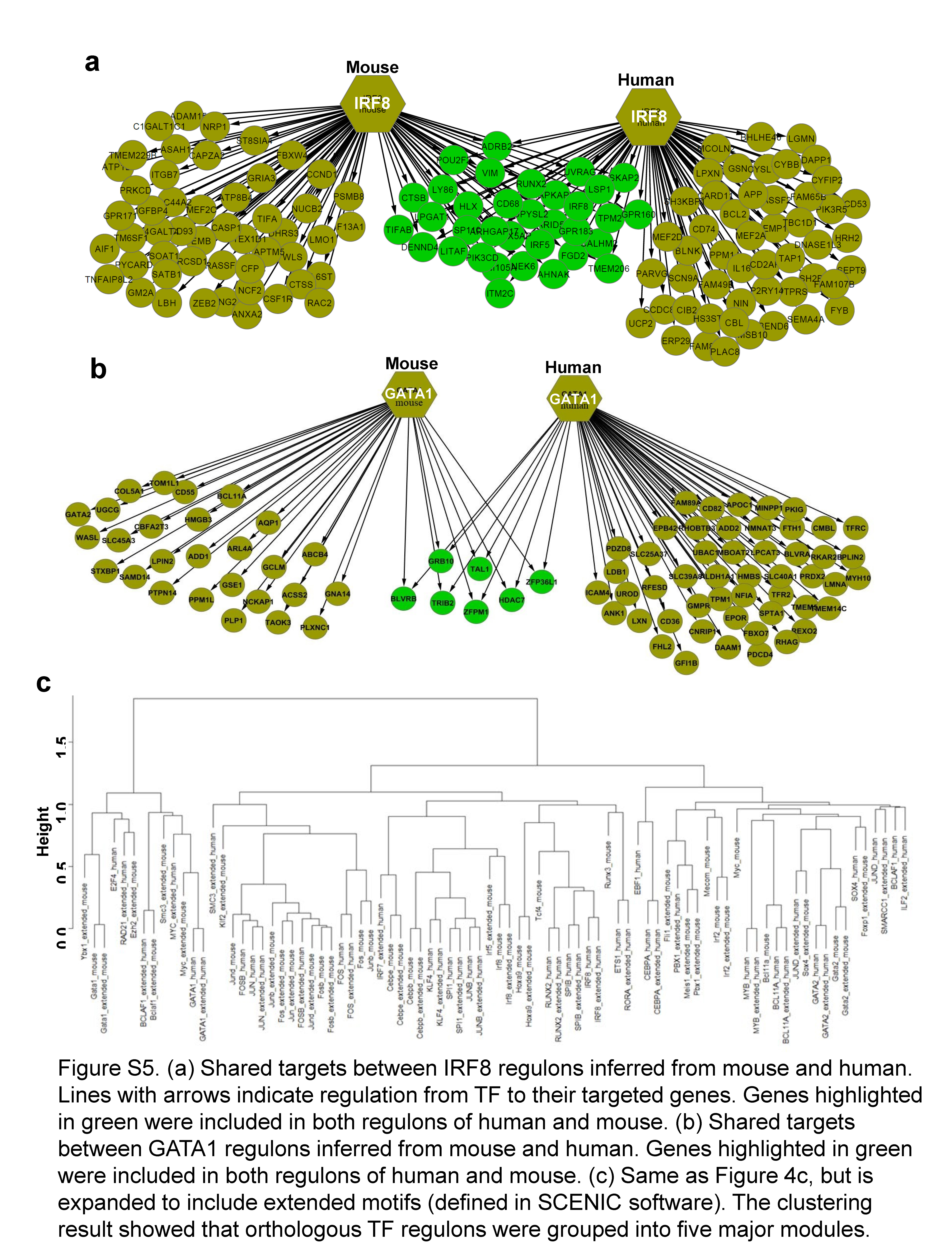

Supplement: Supplementary file 1 [file cells-10-00973-s001.zip › supplementary materials/Fig_S5.jpg]

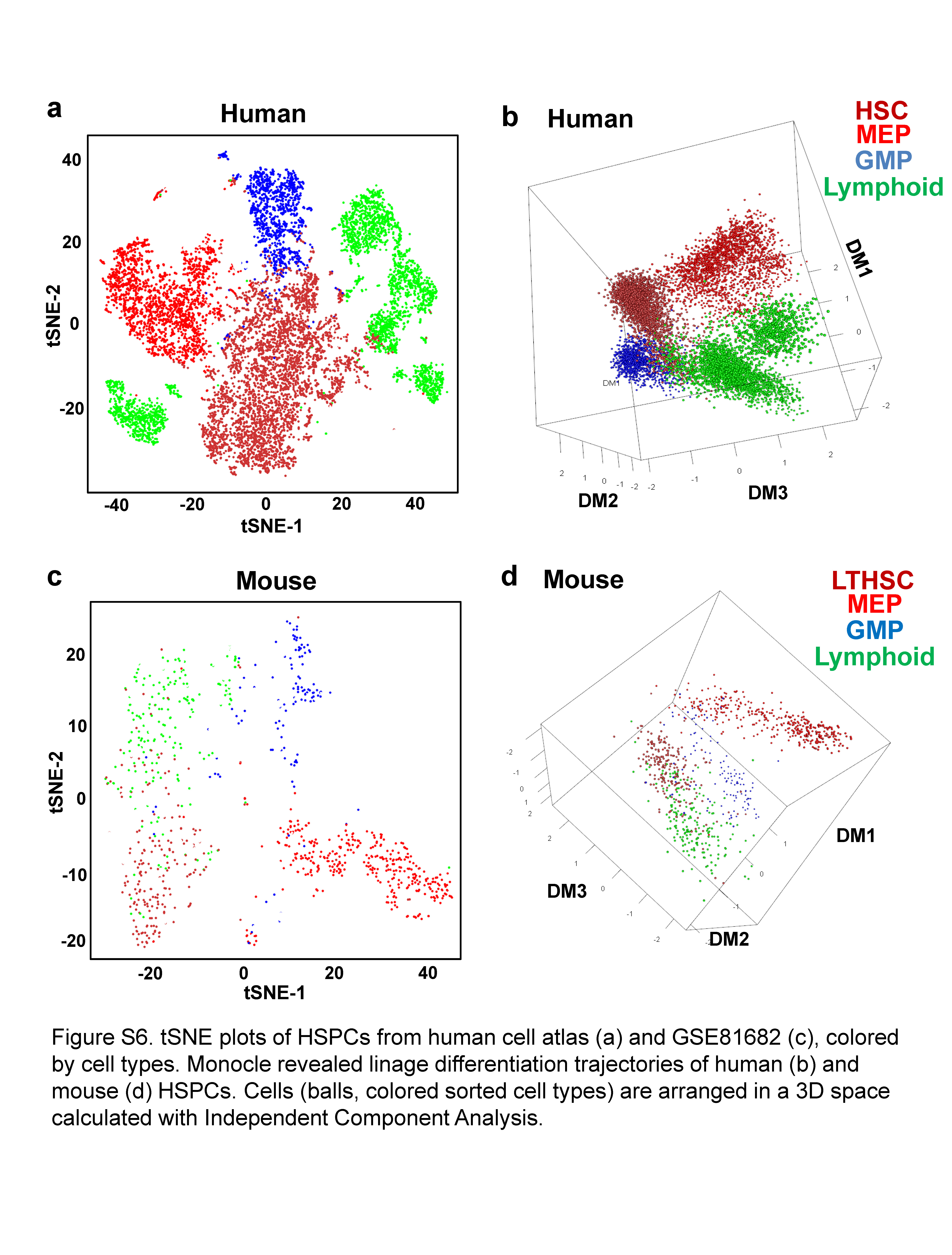

Supplement: Supplementary file 1 [file cells-10-00973-s001.zip › supplementary materials/Fig_S6.jpg]
